# p38 MAPK Endogenous Inhibition Improves Neurological Deficits in Global Cerebral Ischemia/Reperfusion Mice

**DOI:** 10.1155/2022/3300327

**Published:** 2022-06-29

**Authors:** Kun Hou, Zhi-cheng Xiao, Hai-Long Dai

**Affiliations:** ^1^Key Laboratory of Cardiovascular Disease of Yunnan Province, Clinical Medicine Center for Cardiovascular Disease of Yunnan Province, Department of Cardiology, Yan'an Affiliated Hospital of Kunming Medical University, Kunming 650500, China; ^2^Yunnan Key Laboratory of Stem Cell and Regenerative Medicine, Institute of Molecular and Clinical Medicine, Kunming Medical University, Kunming 650500, China; ^3^Department of Anatomy and Developmental Biology, Monash University, Clayton 3800, Australia

## Abstract

Cerebral ischemia/reperfusion (I/R) injury is a complex pathophysiological process that can lead to neurological function damage and the formation of cerebral infarction. The p38 MAPK pathway has attracted considerable attention in cerebral I/R injury (IRI), but little research has been carried out on its direct role in vivo. In this study, to observe the effects of p38 MAPK endogenous inhibition on cerebral IRI, p38 heterozygous knockdown (p38^KI/+^) mice were used. We hypothesized that p38 signaling might be involved in I/R injury and neurological damage reduction and that neurological behavioral deficits improve when p38 MAPK is inhibited. First, we examined the neurological damage and neurological behavioral deficit effects of I/R injury in WT mice. Cerebral I/R injury was induced by the bilateral common carotid artery occlusion (BCCAO) method. The cerebral infarction area and volume were assessed and analyzed by 2,3,5-triphenyltetrazolium chloride (TTC) staining. p38 MAPK and caspase-3 were detected by western blotting. Neuronal apoptosis was measured using TUNEL staining. Neurological deficits were detected by behavioral testing. Furthermore, to assess whether these neuroprotective effects occurred when p38 MAPK was inhibited, p38 heterozygous knockdown (p38^KI/+^) mice were used. We found that p38 MAPK endogenous inhibition rescued hippocampal cell apoptosis, reduced ischemic penumbra, and improved neurological behavioral deficits. These findings showed that p38 MAPK endogenous inhibition had a neuroprotective effect on IRI and that p38 MAPK may be a potential therapeutic target for cerebral IRI.

## 1. Introduction

Ischemic stroke is a serious threat to human life, it is the third leading cause of human death, and nearly 87% of strokes are caused by ischemia, but there are few effective treatments [1, 2]. Cerebral ischemia-reperfusion injury (IRI) refers to brain cells that are damaged after cerebral ischemia and very commonly occurs in cerebral infarction, shock, and suffocation caused by low blood-brain perfusion in clinical practice. IRI is generally thought to be another significant clinical problem in the treatment of cerebral damage, and the recovery of blood reperfusion cannot only lead to the recovery of tissue and organ function; in contrast, the phenomenon that further increases the weight of ischemic injury is not yet completely understood [3].

Global cerebral ischemia has been reported to induce forebrain or whole-brain transient ischemia which can induce neuron loss in the hippocampal CA1 region [4]. With the increase in the occurrence rate of ischemic cerebrovascular disease, animal models have recently become a widespread concerned. The bilateral common carotid artery occlusion (BCCAO) modeling method can be used to study transient global ischemia, has also been proposed as a model of vascular dementia, and has been widely used in recent years [5]. Cerebral IRI is a more complex mechanism involved in the pathophysiological process, and neuronal apoptosis is one of the most common forms of injury and the process of apoptosis by gene regulation. It may also be linked to multiple behavioral and psychiatric changes and has usually been seen following stroke [6]. Although IRI has received considerable critical attention and has some progress in comprehending the mechanisms, there has been little established targeted therapy to safely and significantly lessen its effects [7]. There is a need to develop new prevention and treatment strategies for cerebral IRI.

Mitogen-activated protein kinase (MAPK) signal transduction pathways, such as the p38 pathway and the c-Jun N-terminal kinase (JNK), have been focused on cerebral ischemia/reperfusion. Mitogen-activated protein kinase p38 (p38 MAPK) is a key factor in various physiological processes such as cell growth, propagation, differentiation, death, and intercellular function. The p38 MAPK pathway has attracted considerable attention in IRI, but little research has been carried out on its direct role in vivo. In this study, to observe the effects of p38 MAPK endogenous inhibition on cerebral IRI, we hypothesized that p38 MAPK endogenous inhibition can reduce neurological deficits, neuronal apoptosis, and infarct volume in a cerebral IRI mouse model. To test this hypothesis, we examined the effects of BCCAO on neuronal degeneration, infarction area, hippocampal volume, neurological deficits, and the expression of p38 MAPK and caspase-3 in the hippocampus in wild-type mice and p38 heterozygous knockdown (p38^KI/+^) mice.

## 2. Materials and Methods

### 2.1. Animals and Experimental Procedures

C57BL/6 wild-type mice and p38^KI/+^ mice were obtained from the Institute of Molecular and Cell Biology (Biopolis Drive, Proteos, Singapore). Transgenic animal models were used for the experiment as previously described [8]. Generation of the p38^KI/+^ mice is by mutation and inactivation of phosphorylation of the Thr180 and Tyr182 sites, which were on the C57BL/6 background. They are fed with sterilized food and water. The animals were housed under controlled conditions: temperature (20-24°C) and humidity (50-60%), kept on a 12 h light/dark cycle. Adult male mice (8–10 weeks old) weighing 24 ± 2 g were used.

Healthy 9-week-old male C57BL/6 WT mice (*n* = 82) and male p38^KI/+^ mice (*n* = 18) were selected and randomly divided into three experimental groups: (1) sham group (*n* = 14): control C57BL/6 mice were exposed to only bilateral common carotid arteries (wild-type (WT) sham group); (2) WT & I/R group (*n* = 68): ischemia/reperfusion in C57BL/6 mice; (3) p38^KI/+^ & I/R group (*n* = 18): ischemia/reperfusion in p38^KI/+^ mice. All experimental procedures were reviewed and approved by the Ethics Committee of Kunming Medical University. In this study, all efforts were made to reduce the total number of animals used and minimize experimental animal suffering by following this principle. All experimental procedures were conducted following our Institutional Guidelines for Animal Research and the Guide of NIH (NIH Publication No. 85–23, revised 2011).

### 2.2. Surgery for Induction of Ischemia and Reperfusion

I/R-induced cerebral ischemia and reperfusion injury were induced in mice by bilateral common carotid artery occlusion (BCCAO), as previously methods described [9, 10]. Adult male mice weighing 25 ± 3 g were used. Briefly, mice were anaesthetized with intraperitoneal administration of pentobarbital sodium (1.0%, 80 mg/kg). Then, both vertebral arteries of mice were occluded permanently by electrocautery, both carotid arteries were isolated, and it was made to expose the common carotid arteries by a ventral midline incision. The vagus nerves were carefully separated. Two 3-0 sutures were hobbled in the common carotid arteries with temporarily termination of the blood circulation. At 20 min after occlusion, the sutures were removed, and the blood circulation was restarted. The same was done for the sham-operated control group except the occlusion of the two carotids. To be ready to react quickly and efficiently to surgery recovery for the mice, we used an electric heater (maintained at 36.5-37.5°C) after the operation.

### 2.3. Western Blot Analysis

Western blot experiments were performed as previously described in our lab [8]. The hippocampus was rapidly separated, and homogenates were lysed in RIPA buffer (Merck Millipore). Lysates from hippocampi were dissolved and then boiled in sample buffer (Bio-Rad) at 95°C for 5 minutes. Equal amounts of lysates were loaded on SDS-PAGE gels and transferred to PVDF membranes (Merck Millipore). The PVDF membranes were blocked in TBS-T containing 5% fat-free milk for 1 h. After blocking, the blots were incubated with the primary antibodies overnight at 4°C. This study used the following primary antibodies, including rabbit anti-p-p38 (1 : 1000, Cell Signaling Technology), rabbit anti-c-caspase-3 (1 : 1000, Cell Signaling Technology), rabbit anti-p38 (1 : 1000, Anbo), rabbit anti-caspase-3 (1 : 1000, Abcam), and *β*-actin (1 : 2000, Sigma). After washing, secondary antibodies were incubated for one hour at room temperature. The blots were processed and analyzed quantitatively using Image Lab-5.2.1 software (Bio-Rad).

### 2.4. Brain Infarct Area and Infarct Volume Measurements

The cerebral infarction area and volume were analyzed by using the 2,3,5-triphenyl tetrazolium chloride (TTC) staining method. At 24 h after ischemia and reperfusion, the animals were anesthetized and brain tissue was obtained. To detect the infarct area, the brains were sliced into 2 mm thick coronal sections and then incubated with TTC (Sigma–Aldrich) solution (2%) at 37°C for 10 min in the dark. The infarct area was calculated using the following formula: reduced area = [(contralateral–ipsilateral)/contralateral] 100%. IPP (Ver. 6.0) software was used for infarct volume analysis [11].

Each brain slice was photographed, and the bilateral areas were analyzed with IPP (Ver. 6.0) software. The reduced brain area of each brain slice, which was in the ipsilateral hemisphere compared with the contralateral side, was calculated by the formula. To clarify the difference in brain size, the ratio of hemisphere area, which is the brain contralateral hemisphere, was determined. Infarct volume was calculated as *V* = *t* × (*A*_1_ + *A*_2_+⋯*A*_*n*_)/2 − *t* × (*A*_1_ + *A*_n_)/2 mm^3^, where *A*_1_ to *A*_*n*_: it presented each slice area from 1 to *n* mm^2^. The infarct volume was calculated by formula = (infarct volume × contralateral volume)/ipsilateral volume. This method was modified from previous publications and is described below for references [11, 12].

### 2.5. TUNEL Staining

Apoptotic cells in the brain were detected by the TUNEL staining method. The brain was sectioned at 16 *μ*m thickness (Leica DM2500) and stained with a TUNEL assay kit (Roche) in a dark humidified chamber for 1 h at 37°C. After the TUNEL reaction mixture, it was to make a wash for 10 min three times using PBS buffer. Then, the sections were stained with diaminobenzidine (DAB) (Cell Signaling) substrate solution. Finally, it was viewed and analyzed. The positive apoptotic cells showed a brown stain within the cytoplasm. Concerning each experiment, nonspecific labeling was examined by omitting TdT. The cell numbers of TUNEL-positive neurons and total neurons were analyzed by counting six random areas of sections in the hippocampal CA1 layer. The sections were examined under a Leica fluorescence microscope (400x magnification). Neuronal damage was evaluated and calculated, and the percentage of TUNEL-positive neurons/the number of total neurons was utilized to quantify neuronal damage [13, 14].

### 2.6. Behavioral Testing Procedures

#### 2.6.1. Neurological Severity Score (NSS)

Double-blind trials, control groups, and experimental groups were not identified by the researchers, only after all data were recorded. The experimental protocol was previously described [15–17]. Motor coordination was evaluated by using a modified NSS that contains balance, motor, and reflexes. A scale of 0-18 was used to assess neurological function. One point was classified as an animal not performing the behavioral test or lack of a reflex. A higher score may indicate more severe injury.

#### 2.6.2. Neurological Deficit Score (NDS)

After the animal wakes up from the model experiment of global cerebral ischemia/reperfusion, neurological deficit score experiments were performed according to the behavioral symptoms [18, 19]. Mouse in experiments group one by one score and total scores were assessed using the single-blind method, namely, the total mouse brain stroke index. Briefly, the animals were placed on a smooth tabletop, behavioral scoring by the presence or absence of the following symptoms: vertical hair, to reduce or slow motion, enhanced alertness of the ear and ptosis of the eyelids were evaluated assigning a score (0 or 1). Subsequently, Alice head bent or bent, closure of eyes, circle the body, hind legs open and convulsion or clonic were evaluated assigning a score (0 or 3). Limb weakness was evaluated assigning a score (0 or 6). Total points range from 0 to 25 score, total score < 10 indicates mild ischemia, and total score ≥ 10 indicates severe ischemia.

#### 2.6.3. The Observation of Neurobehavioral Function

All animals were trained in walking 3 days before the operation and 1 hour before ischemia. The basal values were measured and measured continuously after ischemia times. Walking test: the mouse was observed in the width 1.0 cm and length 30 cm of wood in the walking situation; record the passage of time required [20] (simulated and improved protocol from Research Center for Craniocerebral Injury, University of Virginia, United States).

### 2.7. Statistical Analysis

All data are presented as the mean ± standard deviation (SD). Statistical comparisons between two groups were performed using the independent two-sample *t*-test. The data from multiple groups were analyzed using one-way analysis of variance (ANOVA) or Bonferroni's multiple comparison test. A value of *p* < 0.05 was considered statistically significant. Statistical analysis was performed with SPSS 17.0 (SPSS Inc.).

## 3. Results

### 3.1. Cerebral I/R Caused Activation of p38 MAPK, “Core” Infarction, and Ischemic Penumbra

**Figure 1:**
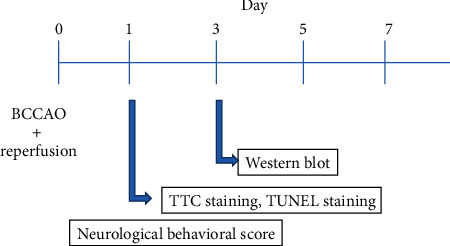
Basic depiction of the experimental design. Global cerebral ischemia/reperfusion treatment at the age of about eight-week-old male mice. In this study, three experimental groups were analyzed: sham group, WT & I/R group, and p38^KI/+^ & I/R group. TTC staining, western blot analysis, TUNEL staining, and neurological score were performed and analyzed.

**Figure 2:**
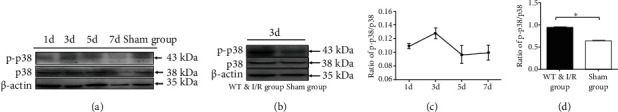
The expressions of p38 phosphorylation and p38 protein are time-dependent. (a) Original blots show the expression of p-p38 in the hippocampus after cerebral ischemia/reperfusion at 1 d, 3 d, 5 d, and 7 d in the WT & I/R group, compared to the sham group. (b) The bar graph shows the quantification of p-p38 MAPK phosphorylation in the hippocampus at 1 d, 3 d, 5 d, and 7 d after cerebral ischemia/reperfusion. (c, d) The expression of p-p38 in the hippocampus at 3 days after cerebral ischemia/reperfusion in WT & I/R and sham groups (*n* = 4, ^∗^*p* < 0.05, sham vs. I/R group). Data are represented as mean with SD. The comparisons were performed by Student's *t*-test. ^∗^*p* < 0.05 versus the control group. Values are mean ± SD. ^∗^*p* < 0.05 by one-way ANOVA.

Basic depiction of the experimental design is in Figure 1. In the experiment, there was some time point (1, 3, 5, and 7 d) identified in C57BL/6 after BCCAO and reperfusion. We found that the expressions of p38 phosphorylation and p38 protein were time-dependent. The level of p-p38 protein in the hippocampus in I/R mice was initially increased and decreased later after cerebral ischemia/reperfusion at 1 d, 3 d, 5 d, and 7 d in the WT & I/R group (Figures 2(a) and 2(b)), and the level of p-p38 protein was significantly higher than in the sham group after 3 days of BCCAO and reperfusion (Figures 2(c) and 2(d), *p* < 0.05).

**Figure 3:**
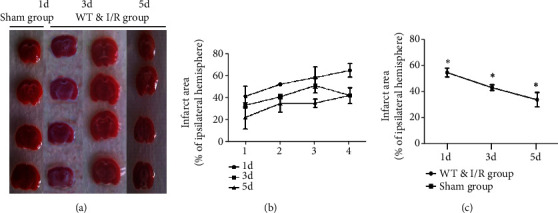
TTC staining was performed at 1 d, 3 d, and 5 d after cerebral ischemia/reperfusion. (a) TTC staining sections graph at 1 d, 3 d, and 5 d cerebral ischemia/reperfusion compared with the sham group. The white color indicates the infarct area. (b) The bar graph is a representation of the quantitative infarct area with TTC staining at 1 d, 3 d, and 5 d. (c) The bar graph represents quantitative infarct volume from the TTC staining at 1 d, 3 d, and 5 d cerebral ischemia-reperfusion, compared with the sham group, *n* = 3 per group, ^∗^*p* < 0.05, sham vs. I/R group. ^∗^*p* < 0.05 by one-way ANOVA.

TTC staining is one of the indicators of cerebral ischemic infarction; TTC staining after cerebral ischemia/reperfusion showed more severe injury and forms “core” infarction and ischemic penumbra at 1 d than 3 d and 5 d infarction (Figure 3(c), *p* < 0.05). These results demonstrate that ischemia/reperfusion caused activation of p38 MAPK, “core” infarction, and ischemic penumbra.

In this paper, we found that p38 MAPK is activated and forms “core” infarction and ischemic penumbra in I/R mice, and these results suggest that BCCAO and reperfusion induce neurological function injury.

### 3.2. p38 MAPK Inhibition Rescues Hippocampal Cell Apoptosis in p38^KI/+^ & I/R Mice

**Figure 4:**
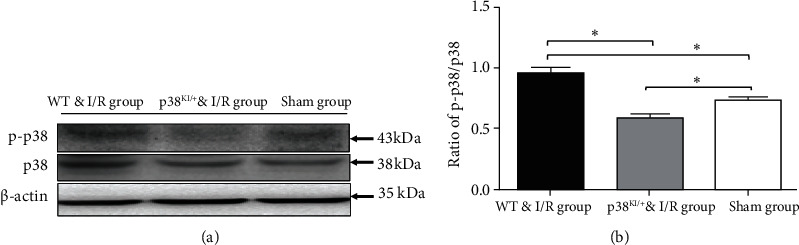
p38 MAPK knockdown reduced p38 MAPK activation in hippocampal CA1 pyramidal cells. (a) Representative images of western blotting in WT and p38 ^KI/+^ mice after BCCAO at 8 weeks of age. (b) Quantification of p-p38 (p38 MAPK phosphorylation) in WT mice in the sham group, the WT & I/R group, and the p38^KI/+^ & I/R group; *n* = 3 mice per experimental group; ^∗^*p* < 0.05, sham group vs. I/R group; ^∗^*p* < 0.05, p38^KI/+^ & I/R group vs. I/R group. ^∗^*p* < 0.05 by one-way ANOVA.

**Figure 5:**
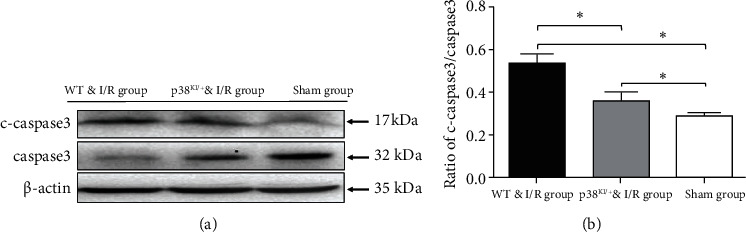
p38 MAPK knockdown reduced the expression of c-caspase-3 in hippocampus CA1 pyramidal cells. (a) Representative images of western blotting in WT and p38 ^KI/+^ mice after ischemia/reperfusion surgery at 9 weeks of age. (b) Quantification of c-caspase-3 activation expression (cleaved-caspase-3/caspase-3) in the hippocampus, mice in the sham group, the WT & I/R group, and the p38^KI/+^ & I/R group. *n* = 3 per group; ^∗^*p* < 0.05, sham group vs. I/R group; ^∗^*p* < 0.05, p38^KI/+^ & I/R group vs. I/R group. ^∗^*p* < 0.05 by one-way ANOVA.

**Figure 6:**
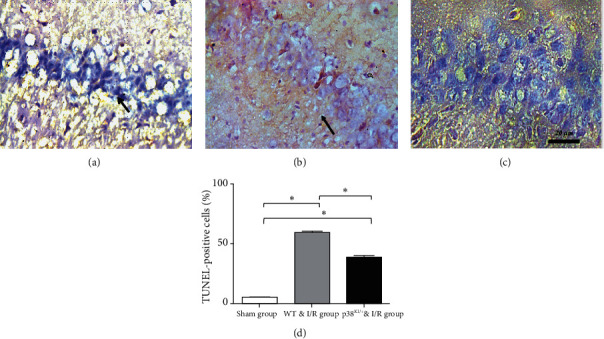
p38 MAPK knockdown rescues apoptosis and DNA damage after cerebral ischemia and reperfusion. (a) TUNEL staining photomicrographs at 3 days after cerebral ischemia/reperfusion in the WT & I/R group. (b) TUNEL staining photomicrographs at 3 d after cerebral ischemia and reperfusion in the p38^KI/+^ & I/R group. (c) TUNEL staining photomicrographs in the sham group by TUNEL staining. (d) Apoptotic TUNEL-positive cells (%) of TUNEL staining in hippocampal CA1; the p38^KI/+^ & I/R group was lower than the WT & I/R group, respectively (*n* = 3 per group; ^∗^*p* < 0.05, sham group vs. I/R group; ^∗^*p* < 0.05, p38^KI/+^ & I/R vs. I/R group.). The slice thickness is 16 *μ*m under a ×400 microscope image (red brown indicates Klenow- or TUNEL-positive cells by black arrows; blue depicts nucleus; scale bars 20 mm). ^∗^*p* < 0.05 by one-way ANOVA.

In this paper, we hypothesized that p38 MAPK inhibition rescues hippocampal cell apoptosis, p38 MAPK, and caspase-3 detected by western blotting in WT mice and p38^KI/+^ & I/R mice after IRI. To confirm that phosphorylation of p38 MAPK critically during ischemia reflected activation, we examined the phosphorylation level of p38 MAPK in the hippocampus. The WT & p38^KI/+^ mice were anaesthetized and subjected to I/R injury mouse model. p38 MAPK and caspase-3 were detected by western blotting. The level of p38 MAPK phosphorylation was analyzed in the hippocampal lysates at 1-day postsurgery. We observed that p-p38 MAPK in the hippocampal lysates was significantly increased in the WT & I/R mice (Figure 4, *p* < 0.05). In contrast, compared with WT & I/R mice, the level of p-p38 was significantly decreased in p38^KI/+^ & I/R mice (Figure 4, *p* < 0.05). The level of cleaved-caspase-3 relative to caspase-3 was a remarkable reduction in the p38^KI/+^ & I/R group, compared with that in the WT & I/R mouse group (Figure 5, *p* < 0.05). TUNEL staining is a common method for detecting DNA fragmentation and widely used to study apoptosis in tissues. The TUNEL-positive cells (%) were increased in the ischemic hippocampal CA1 region in WT & I/R mice (Figures 6(a) and 6(b)). However, the TUNEL-positive cells (%) were less in the ischemic hippocampal CA1 region in p38^KI/+^ & I/R (Figures 6(b) and 6(c)). Apoptotic index of TUNEL staining in hippocampal CA1 area in WT & I/R mice is significantly higher than that in p38^KI/+^ & I/R mice (Figure 6(d), *p* < 0.05). These results showed that p38 MAPK inhibition rescues hippocampal cell apoptosis. p38 MAPK inhibition rescues hippocampal cell apoptosis and reduces ischemic penumbra in p38 ^KI/+^ & I/R mice.

In this paper, upregulation of the expression of phosphorylated p38 MAPK, the cleared of caspase-3 and TUNEL-positive cells, in the hippocampus subregion after reperfusion in WT & I/R mice, the level of phosphorylated p38 MAPK, and the cleared of caspase-3 and the TUNEL-positive cells were markedly decreased after ischemia in p38^KI/+^ mice.

### 3.3. p38 MAPK Inhibition Can Reduce Ischemic Penumbra and Improve the Neurological Behavioral Deficit

**Figure 7:**
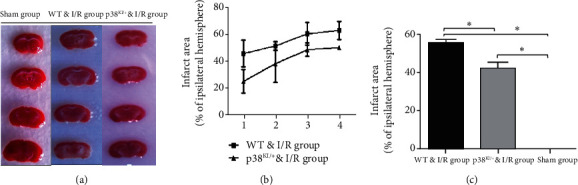
p38 MAPK knockdown rescues brain injury after cerebral ischemia and reperfusion. (a) TTC staining sections graph at 1 d cerebral ischemia-reperfusion in the sham group, the p38^KI/+^ & I/R group, and the WT & I/R group. The white color of sections means the infarct area. (b) The bar graph indicates quantitative infarct area with the TTC staining at 1 d in the sham group, the p38^KI/+^ & I/R group, and the WT & I/R group. (c) Quantitative infarct volume with the TTC staining at 1 d cerebral ischemia-reperfusion in the sham group, the p38^KI/+^ & I/R group, and the WT & I/R group; *n* = 3 per group; ^∗^*p* < 0.05, sham group vs. I/R group; ^∗^*p* < 0.05, p38^KI/+^ & I/R group vs. I/R group. ^∗^*p* < 0.05 by one-way ANOVA.

In this study, we hypothesized that p38 MAPK inhibition reduces brain injury and improves neurological function after cerebral ischemia and reperfusion. TTC staining and neurological behavior were used to evaluate WT mice and p38^KI/+^ & I/R mice after IRI. TTC staining was used to evaluate the improvement of neurological deficits associated with reduced infarct size. In the p38^KI/+^ & I/R group, the TTC staining results showed cerebral ischemia “core” infarction and ischemic penumbra was reduced and ischemic infarction volume markedly decreased than in the WT & I/R group (Figure 7(d), *p* < 0.05). These results showed that p38 MAPK endogenous inhibition improved neurological deficits.

**Figure 8:**
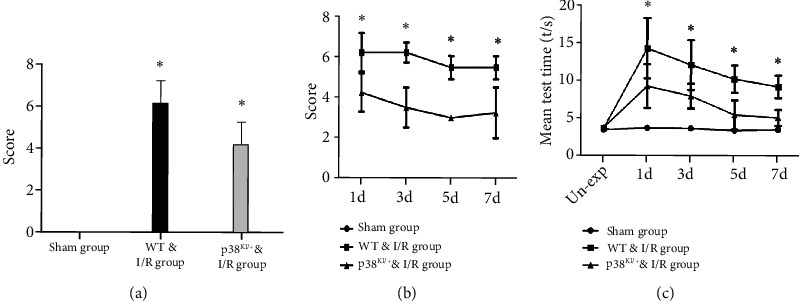
p38 MAPK knockdown improved neurobehavioral function. (a) Neurological severity score at 45 min after reperfusion in the mice of the p38^KI/+^ & I/R group, the WT & I/R group, and the sham group; *n* = 3 per group; ^∗^*p* < 0.05, p38^KI/+^ & I/R vs. I/R group. (b) Neurological deficit score at 1 d, 3 d, 5 d, and 7 d after reperfusion in the mice of the p38^KI/+^ & I/R group, the WT & I/R group, and the sham group; compared with the sham group, *n* = 3 per group, ^∗^*p* < 0.05, p38^KI/+^ & I/R vs. I/R group. (c) The Virginia score after reperfusion was 1 day, 3 days, 5 days, and 7 days in the p38^KI/+^ & I/R group, the WT & I/R group, and the sham group; mean test time reflects neurobehavioral function. *n* = 3 per group; ^∗^*p* < 0.05, sham group vs. I/R group; ^∗^*p* < 0.05, p38^KI/+^ & I/R vs. I/R group. Un-exp represents to have no experiment. ^∗^*p* < 0.05 by one-way ANOVA.

Neurological severity scores (NSS) and neurological deficit scores (NDS scores) were used to determine motor coordination, balance, motion, and reflection. A series of neurological function scores were performed; it has consistently been found that behavioral function was improved in the p38^KI/+^ & I/R groups, compared to the WT & I/R group (Figures 8(a)–8(c), *p* < 0.05). In this study, p38 MAPK inhibition has the potential to rescue and improve neurological function in a mouse model of I/R. These results demonstrated that p38 MAPK inhibition attenuated ischemic brain injury and improved neurological behavioral deficits.

## 4. Discussion

Ischemic cerebrovascular disease is one of the main neurodegenerative disorders and causes of death and disability. Reperfusion is currently the most effective way to restore blood supply to the ischemic part of the brain. However, cerebral ischemia-reperfusion is also very likely to cause injury to brain tissue, which is known as ischemia-reperfusion injury (IRI). IRI is a physiological and pathological process involving multiple mechanisms. Global cerebral ischemia has contributed to the study of the chronic cerebral hypoperfusion effect on cognitive dysfunction and neurodegenerative processes [21–24]. The bilateral common carotid artery occlusion (BCCAO) modeling method can be used to study transient global ischemia [5]. Apoptosis is one of the major results of cerebral IRI and may be induced to generate oxygen free radicals, leading to the process of cell death [25, 26]. Some studies have demonstrated that it leads to a proper reduction in blood flow, causing the cell subtle morphological, biochemical, and behavioral changes and so on [27–30]. BCCAO mice have been found to exhibit long-lasting functional deficits after validation behavioral tests, such as the elevated zero maze (EZM) and the open field test (OF) [31, 32].

The p38 pathway may play important roles in various stages of cerebral ischemia and is involved in some biological responses, such as cell proliferation and apoptosis [33–36]. In the experiment, we found that the expression of p38 phosphorylation and p38 protein was time-dependent. Some studies have shown that the activation of p38 MAPK in furtherance of neuronal death after brain ischemic [37, 38]. In our study, we found that p38 MAPK is activated in the hippocampus of I/R mice. It has been demonstrated that activation of p38 MAPK could defend brain endothelial cells against apoptosis in stroke [39] and protect neurons from ischemic stimulation [40, 41]. In our study, we used the BCCAO modeling method and found that global brain ischemia induces sensory, motor, and neurological behavioral function injury and forms a “core” infarction.

In our study, we hypothesized that p38 MAPK inhibition rescues hippocampal cell apoptosis, p38 MAPK, and caspase-3 detected by western blotting in WT mice and p38^KI/+^ & I/R mice after IRI. We observed that p-p38 MAPK in hippocampal lysates led to a significant increase in the I/R mice compared to WT mice. In contrast, there was a significant decrease in p38^KI/+^ & I/R mice in comparison to WT & I/R mice. In a previous study, p38 MAPK might regulate caspase-3 activation, and it was revealed that p38 MAPK can be directly phosphorylated, causing the inhibition of caspase-3 activity and leading to apoptosis via direct molecular interaction [42]. It has been demonstrated that caspase-3 activation ultimately leads to cell death [43]. We found that the protein level of cleaved-caspase-3 relative to caspase-3 was remarkably reduced in the p38^KI/+^ & I/R group compared with the WT & I/R mouse group. TUNEL staining was utilized for the quantification of neuronal damage [13, 14]. In this study, the expression of phosphorylated p38 MAPK, the cleared of caspase-3, and the number of TUNEL-positive cells increased in the hippocampal subregion after reperfusion in WT & I/R mice, and the levels of phosphorylated p38 MAPK, the cleared of caspase-3, and the number of TUNEL-positive cells were markedly decreased in p38^KI/+^ & I/R mice. These results showed that p38 MAPK endogenous inhibition rescues hippocampal cell apoptosis.

In this study, we hypothesized that p38 MAPK inhibits brain injury and improves neurological function after cerebral ischemia and reperfusion. TTC staining and neurological behavior were evaluated in WT mice and p38^KI/+^ & I/R mice after IRI. In mice with BCCAO-induced brain ischemia-reperfusion damage, TTC staining was used to evaluate brain injury [44, 45]. In the p38^KI/+^ & I/R groups, the TTC staining results showed cerebral ischemia “core” infarction and ischemic penumbra diminution, and the ischemic infarction volume was markedly decreased compared with that in the WT group after cerebral ischemia-reperfusion. NSS and NDS scores were used to determine motor coordination, balance, motion, and reflection function [18, 19]. Neurological scores were used to induce cerebral injury by the BCCAO method. The effect of OKLE (O. kilimandscharicum leaf extract) on motor coordination was evaluated using modified neurological severity scores (NSS) [45], which are a combination of balance, motor, and reflexes. A score of 0-15 was used to score neurological function, and one score was awarded to animals for not performing the test or lack of a reflex. Thus, a higher score indicates more injury [46]. In the p38^KI/+^ & I/R groups, a series of neurological function scores were calculated, and behavioral function was consistently improved compared with that in the WT & I/R group. These results demonstrate that p38 MAPK inhibition attenuated ischemic brain injury and improved neurological behavioral deficits. In our study, we found that cerebral ischemia “core” infarction, ischemic penumbra was diminished, ischemic infarction volume markedly decreased, and behavioral function was improved in the p38^KI/+^ & I/R groups, compared to WT & I/R mice. These results suggest that p38 MAPK endogenous inhibition can reduce ischemic brain injury and improve neurological behavioral deficits.

Previous studies have confirmed the efficacy of p38 inhibitors by the downregulating of p38 MAPK, which is reported to have neuroprotective effects that can reduce the extent of cerebral ischemia, reduce the loss of neurons [37, 47, 48], and improve LV function [49]. However, the current study of the protective effects of this pathway in cerebral ischemia is unclear. In other studies, it has been reported that SB203580 was irrelevant to p38 MAP kinase activity [50]. It is noteworthy that the activation of p38 expression in large mammal animal models, for example, pigs and dogs, was not as pronounced as in rodents [51]. In our lab, we studied the inhibitory effect of phosphorylation of p38 MAPK with SB203580 and did not find a more obvious reduction in WT mice (data not shown). Some inconsistencies may also be present in large animal models [52]. For these inconsistencies, there is a possible explanation, that is, they are different kinds of signal transduction pathways.

p39 is a regulator of Cdk5 (cycle-dependent kinase 5) in neurons; it exists in synapses, which can regulate neurite growth and may play a role in synaptogenesis [53–55]. Cdk5 controls multiple cellular events in postmitotic neurons and participates in neuronal diseases, including stroke and neurodegenerative diseases [56–59]. In this study, our research only focuses the effects of p38 MAPK in IRI in the brain. In the future, the role of p39 MAPK and other signal pathways in IRI should be studied. In our study, it was used to verify that the p38 MAPK pathway is involved in brain IRI and p38 MAPK endogenous inhibition had a neuroprotective effect by using a p38 heterozygous knockdown (p38^KI/+^) mouse model. Some studies have shown that p38 MAPK inhibition has neuroprotective effects by inhibiting the inflammatory response and autophagy in cerebral ischemia [60, 61]. Furthermore, we intend to further study the other neuroprotective mechanisms of p38 MAPK inhibition.

There are few reports regarding the p38 MAPK pathway with the direct effect in vivo in cerebral ischemia using transgenic animals. In this study, we demonstrate for the first time that the p38 MAPK pathway is involved in brain IRI based on p38 MAPK endogenous inhibition. Using a p38 heterozygous knockdown mouse (p38^KI/+^) model, our group found that p38 MAPK endogenous inhibition has the potential to rescue and improve neurological function in a mouse model of ischemia and reperfusion. In this study, it suggests novel aspects on the p38 MAPK signal pathway in global ischemia.

Currently, strategies for drug therapy and new treatments are also being studied. It is reported that *Biochanin A* can reduce brain I/R-induced injury by inhibiting p38 MAPK signal transduction, thereby serving as an effective neuroprotective agent [62]. In clinical treatment, tissue plasminogen activator (tPA) can effectively treat stroke only for a small number of stroke patients, and mechanical thrombectomy surgery and thrombolysis therapy are affected by diverse thrombus composition in stroke patients [63–65]. Gene therapy for stroke is a promising and new treatment method for patients who are ineffective in drug treatment and unable to undergo surgery. It has been shown that gene therapy can help self-repair vision after cerebral ischemia injury; it proves that gene therapy is an effective and promising treatment for cerebral ischemia injury in a mouse model [66].

In our study, p38 MAPK endogenous inhibition had a neuroprotective effect in a transgenic mouse model of IRI. Overall, p38 MAPK endogenous inhibition is a new therapeutic strategy and it could be indicated that p38 MAPK is a possible therapeutic target for the treatment of ischemic disease.

## 5. Conclusion

In conclusion, our data showed that p38 MAPK endogenous inhibition has the potential to rescue and improve neurological function in a mouse model of ischemia and reperfusion. This study suggested that p38 MAPK may be a possible therapeutic target for IRI. However, in the future, the role of p39 MAPK and other signal pathways in IRI should be studied.

## Data Availability

According to reasonable requirements, the supporting data of this study could be obtained from the corresponding author.

## Supplementary Materials

Supplementary Materials Information table concerning compounds, antibody sources, and figures of the number of animals used and the mortality rate after I/R.
