# Supplementary material for: p38 MAPK Endogenous Inhibition Improves Neurological Deficits in Global Cerebral Ischemia/Reperfusion Mice
Source: Neural Plast. 2022 Jun 29;2022:3300327. doi: 10.1155/2022/3300327 (PMC9259354; doi:10.1155/2022/3300327)
Supplement: Supplementary Materials — Information table concerning compounds, antibody sources, and figures of the number of animals used and the mortality rate after I/R. [file 3300327.f1.docx]

**Supporting date**

**1. Information concerning compounds and antibody sources**

Primary anti-body

| Antibodies | Source | application | manufacturers |
| --- | --- | --- | --- |
| p-p38 | Rabbit | 1:1000 | Cell Signaling Technology |
| p38 | Rabbit | 1:1000 | Anbo |
| c-caspase3 | Rabbit | 1:1000 | Cell Signaling Technology |
| caspase3 | Rabbit | 1:1000 | Abcam |
| β-actin | Mouse | 1:2000 | Sigma |

Secondary anti-body

| Antibodies Source | application | manufacturers |
| --- | --- | --- |
| Goat Anti- Rabbit | 1:2000 | Sigma |
| Goat Anti- Mouse | 1:2000 | cwbiotech |

Compounds information

| compounds | manufacturers |
| --- | --- |
| RIPA buffer | merck millipore |
| SDS | Sigma |
| β- mercaptoethanol | AMRESCO |
| Ammonium persulfate | AMRESCO |
| TEMED | Sigma |
| glycine | Sangon biotech |
| TTC | Sigma |
| DAB | Sigma |
| Hematoxylin | Sigma |

**2. The number of animals used and the mortality rate after I/R**

There were 82 male C57BL/6 mice and 18 male p38^ki/+^ mice for study, and the weight was about 22-28 g. They were divided into three groups: sham group, I/R group and p38KI/+ & I/R group. There were 59 surviving mice and 14 p38^ki/+^ mice. In the sham group, there was no death of mice. However, the I/R group and p38KI/+ & I/R group showed a significant reduction in survival compared with the sham group (Figure 1).


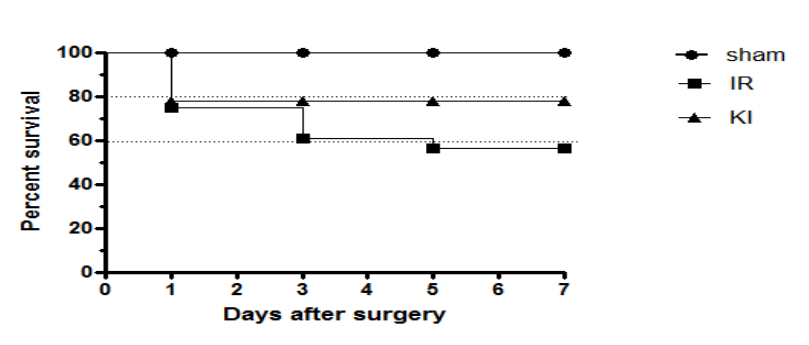


Figure 1: Survival of transient global ischemia during7-day. The transverse axis was the time after the operation, and the longitudinal axis was the time after the operation. Survival rate. Corresponding to the experimental group (n = 14,68,18 in sham, I/R, and p38^ki/+^ I/R group). (ROC survival curve)

Sham, sham group; IR, ischemia/reperfusion group; KI, p38KI/+ & I/R group.
